# Complex spatio-temporal structure of the Holocene Thermal Maximum

**DOI:** 10.1038/s41467-022-33362-1

**Published:** 2022-10-03

**Authors:** Olivier Cartapanis, Lukas Jonkers, Paola Moffa-Sanchez, Samuel L. Jaccard, Anne de Vernal

**Affiliations:** 1 CEREGE, Aix Marseille Université, CNRS, IRD, INRAE, Coll. France, Technopole Arbois, 13545 Aix en Provence Cedex 4, France; 2 MARUM - Center for Marine Environmental Sciences, University of Bremen, Leobener Straße 8, 28359 Bremen, Germany; 3 Department of Geography, Durham University, Durham, DH1 3LE UK; 4 Institute of Earth Sciences, University of Lausanne, Géopolis, 1015 Lausanne, Switzerland; 5 Oeschger Center for Climate Change Research, University of Bern, 3012 Bern, Switzerland; 6 Geotop, Université du Québec à Montréal, PO Box 8888, Montréal, QC H3C 3P8 Canada

**Keywords:** Palaeoclimate, Palaeoceanography, Climate and Earth system modelling

## Abstract

Inconsistencies between Holocene climate reconstructions and numerical model simulations question the robustness of climate models and proxy temperature records. Climate reconstructions suggest an early-middle Holocene Thermal Maximum (HTM) followed by gradual cooling, whereas climate models indicate continuous warming. This discrepancy either implies seasonal biases in proxy-based climate reconstructions, or that the climate model sensitivity to forcings and feedbacks needs to be reevaluated. Here, we analyze a global database of Holocene paleotemperature records to investigate the spatiotemporal structure of the HTM. Continental proxy records at mid and high latitudes of the Northern Hemisphere portray a “classic” HTM (8–4 ka). In contrast, marine proxy records from the same latitudes reveal an earlier HTM (11–7ka), while a clear temperature anomaly is missing in the tropics. The results indicate a heterogeneous response to climate forcing and highlight the lack of globally synchronous HTM.

## Abstract (web summary)

Analysis of the largest available database of Holocene temperature time series covering past 12,000 years reveals complex spatio-temporal trends and challenges the paradigm of a globally synchronous Holocene Thermal Maximum.

## Introduction

Natural climate variability results from multiple forcings and feedbacks with heterogenous spatiotemporal manifestations. Greenhouse gases, volcanic radiative forcing, and solar irradiance apply rather homogeneously across the Earth’s surface, while insolation varies both latitudinally and seasonally. In addition, the climate system response may be amplified or dampened by feedbacks inherent to changes in physiography, albedo, and by variations in oceanic and/or atmospheric circulation that (re)distribute heat across the Earth’s surface. Our understanding of climate processes is limited by the rather short temporal span and heterogenous spatial coverage of instrumental records. Evidence of past climate variability gleaned through the testimony of geological archives thus offers a unique opportunity to contextualize ongoing changes and to assess climate model performance on timescales going beyond the decadal climate variability recorded in the instrumental period.

The temperature at the Earth’s surface responds directly to global radiative forcing and thus provides fundamental insights into the state of the climate system. Over the past decades, quantitative indicators of past temperature (hereafter called “proxies”) based on different types of archives have been used to reconstruct climate variability over a range of timescales. The improvement of both spatial coverage and temporal resolution of temperature proxy records led to the development of regional and global temperature reconstructions, which have allowed the scientific community to highlight the unprecedented nature of anthropogenic climate change across the common era^1,2^ and the Holocene^3–6^. Global temperature reconstructions consistently depict a Holocene Thermal Maximum (HTM) typically ranging between 10 and 5 ka^4,5^ with a maximal probability centered around 6.45 ka^4^. The HTM was followed by global cooling until the end of the nineteenth century CE, interrupted by rapid and sustained warming characterizing the industrial era towards the present. Yet, the cooling trend inferred from proxy records, often attributed to declining high northern latitude insolation, cannot be resolved in numerical simulations^7^. Indeed, in climate models, the simulated global mean temperature is predominantly driven by the ice-sheet extent and atmospheric greenhouse gas concentrations, which in synergy impose continuous warming over the course of the Holocene^7^.

This discrepancy between proxy data and model simulations, commonly referred to as “The Holocene Temperature Conundrum”^7^, casts doubt on the conceptual framework underlying temperature proxy interpretation and on climate model skill. For instance, it has been suggested that temperature reconstructions may be seasonally biased^7,8^ and/or that the global mean value is skewed because of the overrepresentation of northern North Atlantic sea-surface temperature (SST) records^5–7^. However, model-data inconsistencies may equally well result from geographically divergent trends due to sea-ice dynamics^9^, polar amplification^10^, insufficient model resolution^11^, and boundary conditions used in numerical simulations^12^. Although the HTM has been intensively studied from a global perspective^3–7^, its spatio-temporal characteristics have received relatively little attention, even though the local and regional trends differ markedly from the globally averaged reconstructions^3,13^.

In this study, we seek to document the spatiotemporal expression of the HTM in the marine and continental realms to shed light on the forcings and feedbacks underpinning the evolution of Holocene climate^9^.

## Results and discussion

### Temperature 12k database analyses

**Fig. 1:**
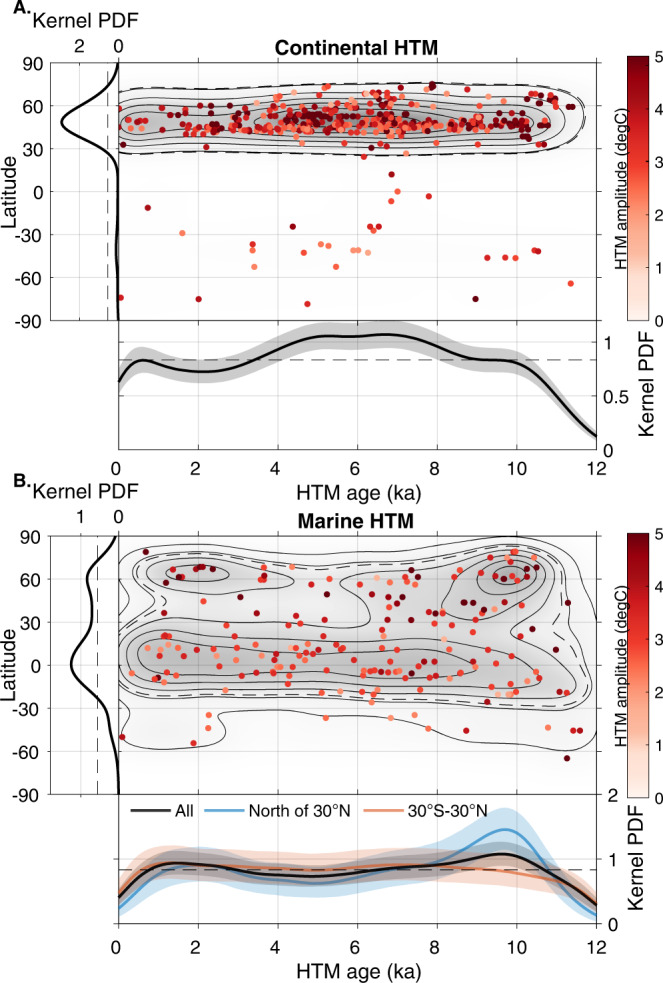
Spatiotemporal structure of the Holocene Thermal Maximum. Spatiotemporal structure of the Holocene Thermal Maximum (HTM) derived from marine and continental temperature records. Dots represent median HTM age and median HTM amplitude (anomaly compared to mean Holocene temperatures) calculated from the age and temperature ensembles for each continental (**A**) and marine record (**B**). Gray shadings and contours depict the mean of 10,000 kernel probability density function maps (PDF; Gaussian kernel with a bandwidth of 10° in latitude and 500 years) calculated using the temperature and age model ensembles (see also Fig. 5 for contour label). Left and bottom panels correspond to median kernel probability density calculated against latitude (10° bandwidth) and age (500 years bandwidth), respectively for different subsets, with dashed straight lines corresponding to a homogenous distribution (color shading shows 10–90 percentiles).

In order to investigate the spatiotemporal expression of the HTM, we analyze the Temperature 12k compilation^3^, which includes 1319 globally distributed paleotemperature records with an average sampling and age control resolution of 164 and 1000 years, respectively. We initially select records that cover at least 80% of the Holocene. This subset includes 233 (184 annual; 32 summer and 17 winter) marine records and 470 (159 annual; 184 summer and 127 winter) continental records (see details in “Methods”). The terrestrial subset is dominated by pollen-based temperature reconstructions from the Northern Hemisphere, with virtually all records (91%) distributed between 40°N and 70°N (Fig. 1a). The SST subset is more widely distributed, although somewhat concentrated towards continental margins and the Northern Hemisphere (Fig. 1b, see details in “Methods”).

**Fig. 2:**
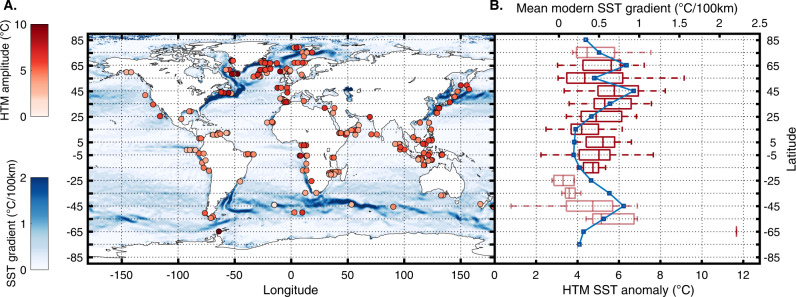
Latitudinal structure of the Holocene Thermal Maximum amplitude. **A** Mean sea surface temperature (SST) gradient^46^ (defined as ${{{{{\boldsymbol{g}}}}}}{{{{{\boldsymbol{=}}}}}}{{{{\parallel }}}}{{{{{\boldsymbol{\nabla }}}}}}{{{{{\boldsymbol{SST}}}}}}{{{{\parallel }}}}$, calculated based on a 0.25° resolution map) and median marine HTM anomalies compared to mean Holocene (dots). **B** Marine Holocene Thermal Maximum anomalies (bottom axis) and mean modern latitudinal SST gradient for 10° latitudinal bands (blue line, top axis). Box-whisker plots show 0% (bottom whiskers), 25, 50, 75, and 100% (top whiskers) quantiles. Semitransparent boxes are for latitudinal bands with fewer than 10 records.

**Fig. 3:**
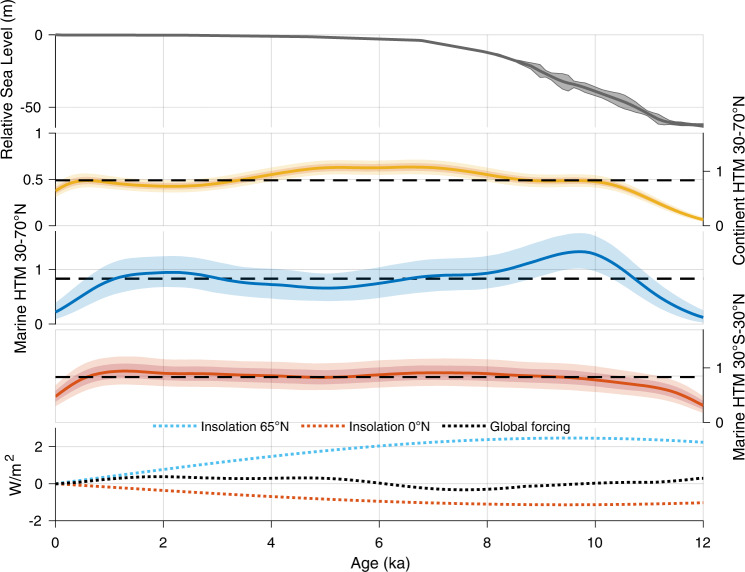
Climate forcing impact on Holocene Thermal Maximum. Relative sea level^47^; Holocene Thermal Maximum (HTM) age probability density function between 30°N and 70°N over land and ocean, and between 30°S and 30°N in the oceans. Mean annual insolation at 65°N and at the equator and the sum of radiative forcing (CO_2_, CH_4_, N_2_O, Volcanic emission and solar irradiance; see details in “Methods”). Orbital forcing were calculated according to refs. 48, 49 using a dedicated function^50^.

**Fig. 4:**
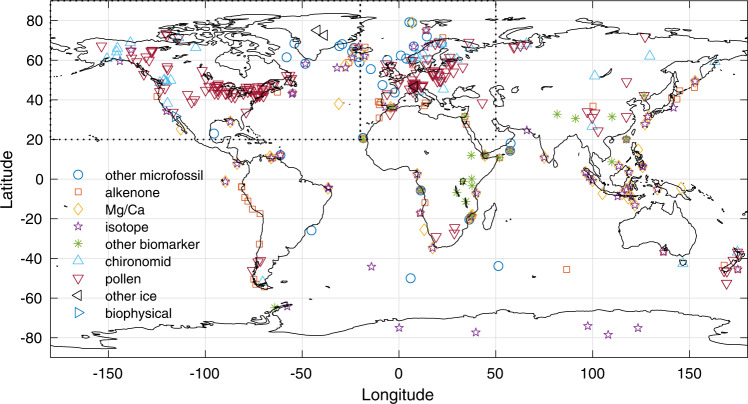
Map of the paleotemperature records. Map of the paleotemperature records selected and used in this study. Dotted lines represent the two domains with high density data used for continental proxy analyses (North America and Western Europe).

**Fig. 5:**
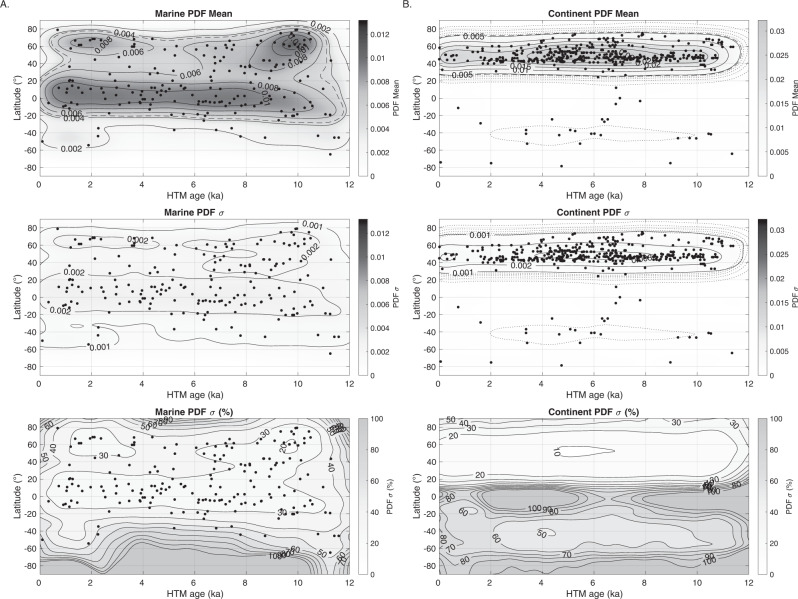
Robustness of the spatiotemporal structure of the Holocene Thermal Maximum. Robustness of the spatiotemporal structure of the Holocene Thermal Maximum for marine (**A**) and continental (**B**) temperature proxy ensembles. Mean, standard deviation (*σ*), and standard deviation (% of local PDF), of the 10,000 PDF maps calculated using ages and temperature ensemble in Temp12k compilation.

**Fig. 6:**
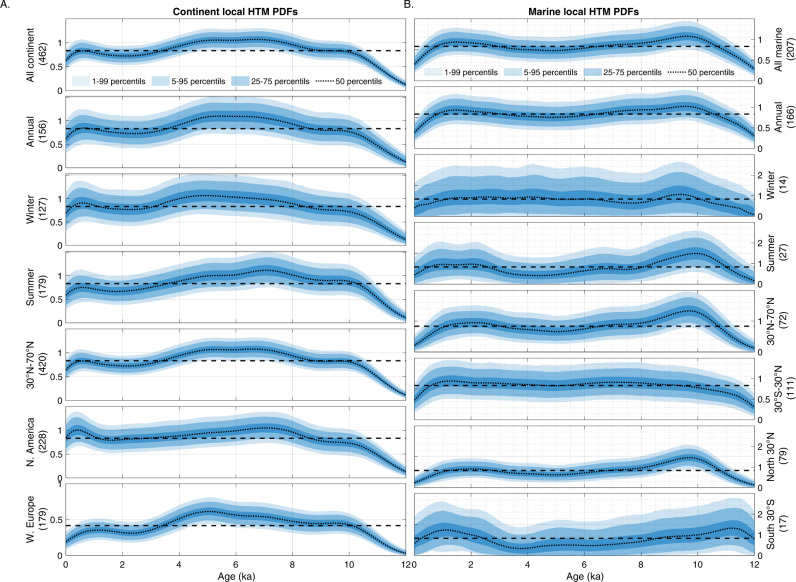
Temporal structure of the local Holocene Thermal Maximums. Temporal structure of the local HTMs for different subsets of continental (**A**) and marine (**B**) proxies. Dotted lines show the median values across 10,000 realizations of the time series. Shading denotes the confidence intervals (1–99, 5–95, 25–75 percentiles, light to dark). The subset used and the number of records available in each subset is shown in the *y* axis label.

**Fig. 7:**
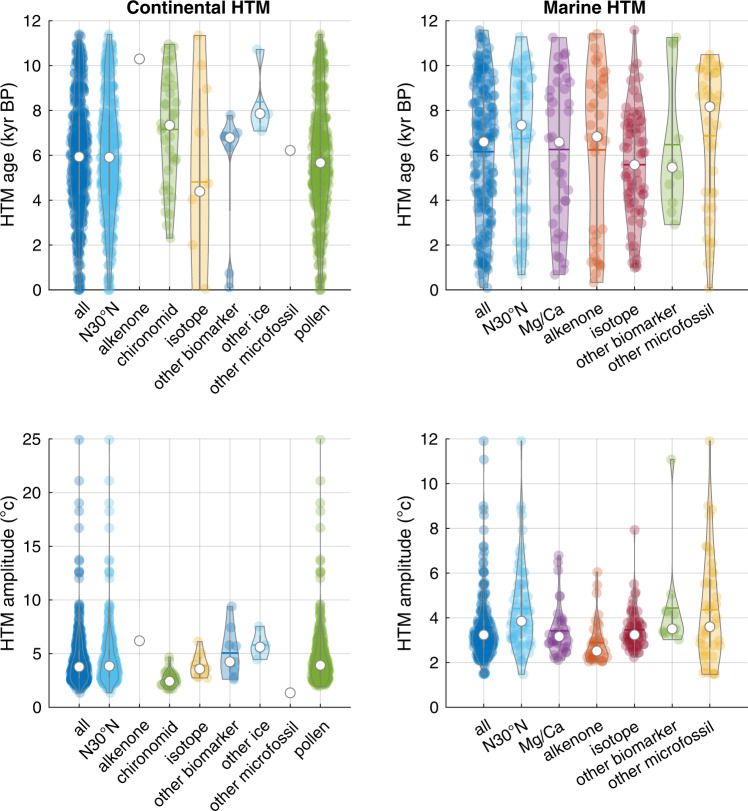
Median HTM ages and amplitudes. Median HTM ages and median HTM amplitudes for marine and continental records depending on the proxies used. Other microfossils include dinocysts, planktonic foraminifera, diatoms and radiolaria; other biomarkers include TEX_86_, GDGTs, BNA15, LDI (see details in ref. 3). The category named “all” includes every records while the”N30°N” category includes all records north of 30°N. The mean (white dot) and median (colored horizontal bar) are displayed for each subset.

The Temperature 12k compilation provides age and temperature ensembles based on dated intervals and proxy calibration (100 members each) for 207 of the selected marine records and for 462 continental records. We use these to assess the robustness of our inferences against reconstruction and chronological uncertainties. We generate 10,000 time series based on the age and temperature ensembles for each record, identify the age of the highest temperature, and calculate the temperature anomaly compared to the mean Holocene value (see details in “Methods”). We subsequently analyze the temporal and spatial distribution of the selected local HTM age ensembles using one- and two-dimensional kernel probability density function (Figs. 1–7, Gaussian kernel, with a bandwidth of 500 years and 10° in latitude, see details in “Methods”).

### Continental HTM

Continental temperature reconstructions between 40°N and 70°N most often reach their maximum values between 8 and 4 ka, with a slight dependence on proxy type (Figs. 1, 5, and 7). The median HTM age based on pollen records (~5.7 ka) lags continental temperature reconstructions based on chironomids (~7.3 ka) by ~1600 years (Fig. 7). Notwithstanding proxy type, the integral of the probability density functions of continental HTM ages between 4 and 8 ka amounts to 40.4%, while representing only 33.3% of the Holocene time interval. In other words, 60% of the records reached maximum temperatures either before or after the 8–4 ka interval, highlighting a dispersion of local HTM ages compared to global reconstructions. The HTM occurs at around 7 ka across North America, but rather later (5 ka) across western Europe, explaining the broad bimodal distribution characterizing the continental records (Fig. 6b). Many of the records are considered to reflect mean annual temperature, while others are assumed to reflect seasonal conditions. However, the probability density function (PDF) of HTM age calculated for winter, summer and annual temperature reconstructions are virtually indistinguishable.

### Marine HTM

Considered globally, marine records do not display a pronounced clustering of HTM ages (Fig. 1). However, the global average eclipses marked spatial variability in HTM timing. Whereas the tropics conform with the global marine reconstruction (30°S–30°N), time series from the mid-high latitudes of the Northern Hemisphere clearly precede the global average (9.7 ka), thus leading the NH continental temperature optimum by ~3500 years (Figs. 1 and 5). Although records south of 30°S remain sparse, they also suggest an early HTM (12–10 ka), hinting at hemispheric symmetry (Fig. 6). As for terrestrial records, the HTM age distributions based on annual and seasonal proxies are undistinguishable, yet the number of winter temperature time series remains insufficient (*n* = 15) to derive statistically robust conclusions (Fig. 6). The HTM anomaly mostly remains below 6 °C and shows a clear meridional pattern with higher temperature amplitudes in the mid latitudes of both hemispheres (Figs. 1, 2, and 7).

### Latitudinal pattern in the expression of the marine Holocene Thermal Optimum

The spatiotemporal structure of the HTM in the marine realm indicates that Holocene temperature trends vary by latitude, with early (11–7 ka) and high amplitude HTM manifestations at mid to high latitudes of the Northern Hemisphere, and muted expression in the tropics, in accordance with previous studies^3,5,13^. The boundary between these climate regimes is located around 30°N (Fig. 1b).

**Fig. 8:**
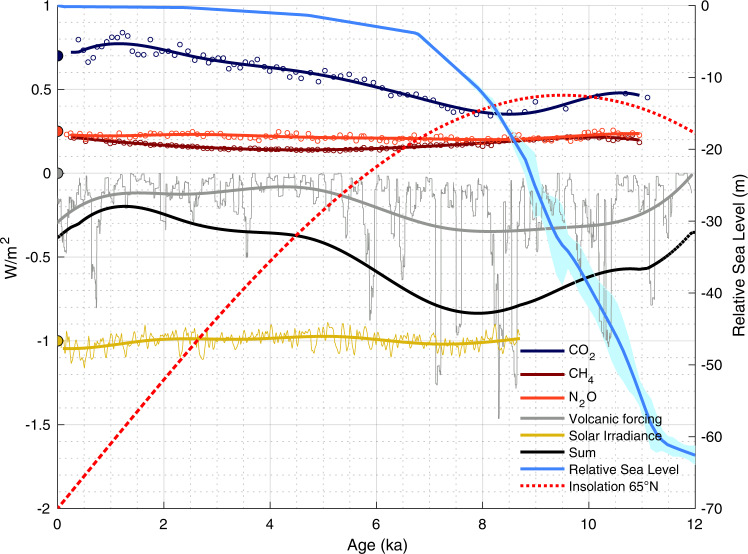
Holocene forcing. Forcing (W/m^2^) due to pCO2^43^; pCH_4_ and pN_2_O^44^; 100-year running mean of volcanic radiative forcing^41^; decadal total solar irradiance variations^42^ at earth surface (ΔTSI at the top of the atmosphere × (1 – albedo)/4, with mean albedo =0.29), mean annual insolation at 65°N^48–50^, and relative sea level^47^. Preindustrial forcing is shown as a filled dot at 0 ka; CH_4_ and N_2_O are superimposed. The sum of radiative forcing (CO_2_, CH_4_, N_2_O, Volcanic and Irradiance) is also displayed. Forcings for CO_2_, CH_4_, N_2_0, and solar irradiance were vertically shifted by 0.7, 0.25, 0.25, and −1, respectively, for better readability of the figure.

**Fig. 9:**
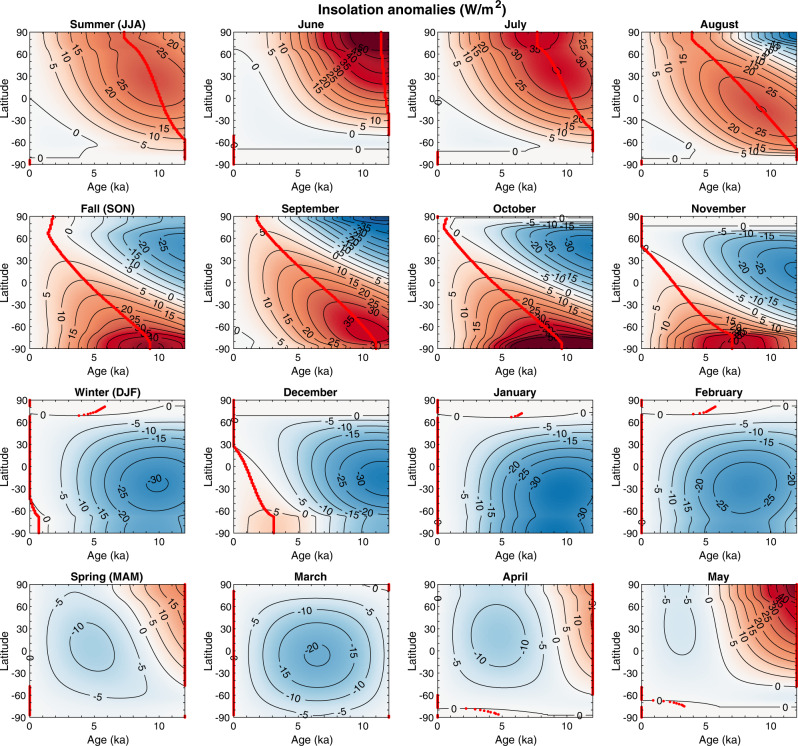
Seasonal and monthly insolation. Seasonal and monthly insolation anomalies compared to modern insolation (W/m^2^) based on orbital parameters^48^ using a Matlab function by Eisenman and Huybers^50^. The red line corresponds to the age of maximal insolation anomaly within 0–12 ka at each latitude.

**Fig. 10:**
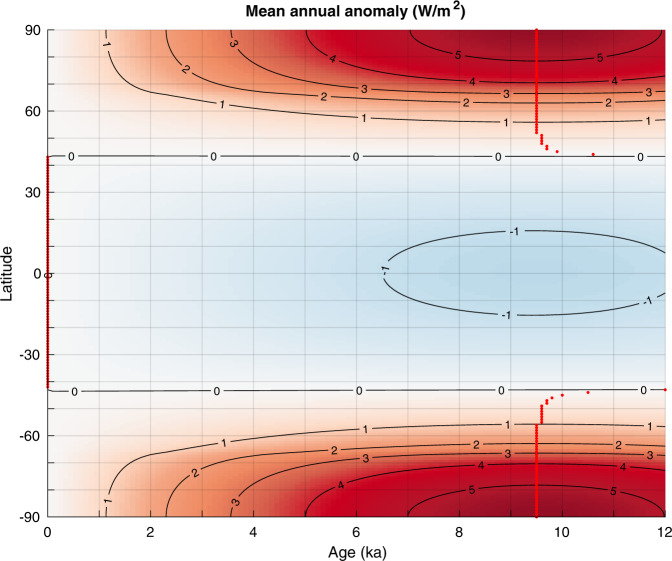
Mean annual insolation. Mean annual insolation anomaly compared to modern insolation (W/m^2^) based on orbital parameters^48^ using the Matlab function by Eisenman and Huybers^50^. The red dots correspond to the age of maximum insolation anomaly within 0–12 ka at each latitude.

The latitudinal pattern in the timing of marine HTM is most likely steered by orbital forcing, because it is the largest contributor to radiative forcing, especially during the early Holocene (Figs. 3, 8–10). However, insolation varies with both latitude and season. The response of the climate system as recorded in proxy timeseries could hence be regionally and temporally variable.

During the Early Holocene mean annual insolation was significantly higher than at present, by up to 5 W/m^2^ near the poles and 2.5 W/m^2^ at 65° (Figs. 3 and 10) but was lower at mid to low latitude (−1 W/m^2^ at the equator). It is therefore conceivable that the zonal trend portrayed by the marine temperature records reflects changes in mean annual insolation. However, forcing with almost similar amplitude in the tropics during late Holocene (2 W/m^2^) due to increasing greenhouse gas concentrations and insolation, was not associated with a clear temperature maximum (Fig. 3). This makes changes in the mean annual forcing alone an unlikely control on the HTM expression. Instead, latitude-dependent mechanisms likely amplified radiative forcing north of 60°N which impacted climate southward to 30°N as a result of polar amplification feedbacks due to sea ice albedo^14^. However, changes in seasonal insolation might offer a more direct explanation of the observed early marine HTM at mid latitudes.

The sensitivity of local mean annual temperatures to seasonal insolation is nonlinear and varies spatially, with most of the tropical to subpolar Earth surface, between 60°S and 60°N, predominantly influenced by summer insolation^15^. During the Holocene, the maximum summer insolation in the Northern Hemisphere occurred between 10 and 7 ka with anomalies of more than 25 W/m^2^ compared to modern (Fig. 9). This is consistent with the inferred early HTM timing in extratropical regions of the Northern Hemisphere (Fig. 3).

The similarity between summer, winter and annual HTM in the marine records evidenced here, despite contrasted seasonal insolation trends, may shed light on the climatic feedbacks affecting local HTM manifestation. While some numerical simulations indicate strong dependency of monthly temperature on monthly insolation, implying divergent seasonal trends over the course of the Holocene^10,16^, other models indicate year-long effects of summer insolation on annual temperature in the ocean^10^ at high- to mid-latitudes. The latter display strong polar amplification, annual temperature dependency on summer sea ice loss and better agreement with annual^10^ and seasonal proxy data (this study), offering a plausible solution to the HTM conundrum. However, it relies on seasonality attributed to proxies which remains imperfectly resolved and debated^8,17,18^. The PDF of the arguably sparser Southern Hemisphere records shows a dual HTM (early and late, Fig. 6) with low confidence. If the interhemispheric symmetry of an early Holocene marine HTM is real despite the asymmetrical seasonal forcing, it must either reflect contrasted sensitivity to seasonal insolation at high latitudes^15^ (potentially due to sea ice dynamics and/or continental configuration), indicate the dominance of high latitude annual insolation forcing over summer insolation on Holocene climate (see above) or suggests the strong global influence of glacier retreat.

The early HTM peak in the Southern Hemisphere probably also relates to changes in the Atlantic Meridional Oceanic Circulation (AMOC) which affected interhemispheric heat transport^13^ through the bipolar seesaw^19^ across the deglaciation, leading to early interglacial conditions (12 ka) in the Southern Hemisphere^20^. The late HTM peak in the SH (Fig. 6) may result from proxy or sites more sensitive to the austral summer insolation that peaked late in the Holocene (Fig. 9). Clearly, a better spatiotemporal coverage of paleotemperature records from the Southern Hemisphere is required to confidently evaluate the relative importance of the aforementioned processes on Holocene climate, in particular the impact of annual and seasonal insolation changes.

### Delayed continental timing of the Holocene Thermal Optimum

Our analysis demonstrates that the HTM occurred earlier in the ocean than on land at the mid latitudes of the Northern Hemisphere, suggesting that different mechanisms and feedbacks drive temperature changes in oceanic and continental realms, or that contrasting biases affect marine and continental temperature proxies.

Continental HTM occurrences between 30 and 70°N peak between 4 and 8 ka, approximately 3.5 kyr later than in the ocean at the same latitudes. Assuming that both marine and continental temperature proxies are not seasonally biased, the comparatively early HTM in the ocean could simply relate to a higher sensitivity of marine temperatures to summer insolation at mid latitudes^15^. In general, the temperature response to seasonal insolation at high latitudes differs in the ocean and on land because of the high thermal inertia of seawater and limited sensitivity to winter insolation due to vertical mixing^15^, not to mention the minimum winter temperature threshold of −1.8 °C that corresponds to the seawater freezing temperature. Hence, the ocean-continent difference could result from a systematic seasonal bias towards summer in the high latitude marine records. However, because of seasonal variability in the abundance of the proxies, both foraminifera-^18^ and alkenone-based temperature reconstructions^21^ are more likely to reflect annual mean conditions at mid-latitudes, whereas pollen-based temperatures are more likely to be biased toward the flowering season (summer)^22^. Because both summer and annual insolation in the Northern Hemisphere peak early in the Holocene, seasonal bias in the proxy records is unlikely to explain the ocean-land difference in the HTM timing.

Marine and continental climate would have also responded differently to climatic feedbacks such as glacier fluctuations, snow cover and vegetation changes^23^. The Eurasian ice sheet disappeared at around 8 ka^24^, while the final deglaciation of the Laurentide ice sheet occurred later at 6.7 ka^25^. The terrestrial HTM coincided with the stabilization of sea level (Fig. 3) when ice sheets receded to their modern limits, suggesting that dwindling continental ice sheets may have delayed warming on land. Although, the HTM occurred slightly earlier close to the Laurentide ice sheet than in Western Europe (Fig. 6), highlighting the complex and spatially heterogenous climatic response to dwindling ice sheets^12,26^.

The generally younger HTM age on land may also partly relate to a specific bias affecting pollen-based temperature estimates. Amongst the continental temperature records, pollen-based records are dominant, and display a generally younger HTM age distribution than the second most abundant continental reconstructions based on chironomid assemblages (Fig. 7). Vegetation changes may not only be modulated by temperature, but also by the hydrological regime, soil development, species migration modes, fire history and anthropogenic pressure among others. For instance, glacial erosion affects the soil formation, particularly at high latitude^27^, leaving a long-lasting imprint of glacial climate on interglacial vegetation^28^. As such, the northward migration and expansion of vegetation during the deglaciation possibly led to a transient disequilibrium between the local vegetation composition, pollen assemblages and climate during the early Holocene. Moreover, changes in vegetation cover at mid latitudes during the deglaciation and the Holocene might have impacted the continental climate through changes in albedo^23,29^ and may have contributed to the time lag with respect to the ocean.

### Latitudinal pattern in the amplitude of the marine HTM

The marine HTM anomaly displays a latitudinal pattern with higher amplitudes centered around 45°S, 45°N and 65°N (Fig. 2). This pattern bears similarity with the pattern in modern SST gradients and the position of oceanic frontal zones (Fig. 2). Today, steep SST gradients occur at the poleward boundary of the oceanic subtropical gyres, around 45° of latitude both in Southern and Northern Hemispheres, and at 65°N, north of the subpolar North Atlantic gyre, close to the gateway between the North Atlantic and the Arctic Ocean (Fig. 2). Thus, the large Holocene temperature anomalies recorded at similar latitudes as modern frontal zones, may reflect enhanced local temperature sensitivity to latitudinal migrations of oceanic fronts and suggests that surface ocean dynamics played a significant role in the recording of the spatiotemporal evolution of Holocene temperatures. High-amplitude variations in SSTs near western boundary currents and frontal zones have been previously recognized^30^. Indeed, the inclusion of records from frontal zones in global reconstructions has been challenged due to their high sensitivity to shifts in surface currents^6,8^. We provide an objective analysis of these features which indicate that a high amplitude HTM is not restricted to western ocean basins, but also affects eastern flanks (Fig. 2). A southward shift of the Gulf Stream and Kuroshio may explain high-amplitude temperature trends near western boundary currents^30^, although an equatorward contraction of the subtropical oceanic gyres is a better candidate in eastern oceanic basins where the spatial temperature gradient is low. Moreover, reduction of the Atlantic Meridional Overturning (AMOC) strength from mid to late Holocene^31^ would have also contributed to cooling^32^. Irrespective of the exact cause of spatial discrepancies, our analysis highlights the importance of small-scale ocean dynamics for the evolution of Holocene seawater temperature. Importantly, numerical simulations from coupled model experiments of mid-Holocene climate exhibit a large dispersion in simulated temperature at frontal zones^33^, likely due to insufficient spatial resolution. This disagreement among simulations corroborates the critical role of oceanic circulation, and the relevance of exploring ocean front dynamics further for simulating long term climate change and addressing the HTM conundrum in more detail.

### Complex Holocene temperature patterns

Our analyses reveal a dynamic and spatially variable evolution of Holocene temperature that contrasts with the notion of a globally synchronous mid Holocene thermal optimum. The observed spatiotemporal structure of the HTM resulted from geographically divergent temperature trends likely related to differential seasonal and spatial responses to climate forcing and feedbacks. The spatiotemporal dynamics should be considered in data-model comparison for the mid Holocene time slice at 6 ka^34^, in particular, the delayed continental HTM on land compared to the ocean, and the latitudinal contrast in the timing and amplitude of the marine Holocene Thermal Maximum we identified here. Proxy records suggest similar annual and seasonal temperature trends both on land and in the ocean across the Holocene. Seasonal trends in the ocean better conforms with numerical simulations displaying a high degree of Arctic amplification due to sea ice loss, which might reconcile proxy-based reconstructions and numerical simulations^10^.

Importantly, the comparison between global mean temperature reconstructions and numerical simulations, at the crux of the HTM conundrum, has limited mechanistic implications. Instead, the spatiotemporal structure of the temperature trends over the Holocene bears more valuable information on the relative importance of the forcing mechanisms and feedbacks acting on climate. The high amplitude temperature variations in frontal zones highlight the importance of oceanic circulation on Holocene climate variability and suggest that model-data (dis)agreement partly hinges on resolving frontal dynamics in climate simulations, in addition to the degree of polar amplification. Gaining confidence in the regional aspects of future climate projections^35^ will therefore partly rely on a better representation of oceanic frontal zones in numeric simulations, but also on improved proxy coverage on land outside the 30°−70°N latitudinal band and in the southern Hemisphere ocean, as well as on development of seasonal proxy records.

## Methods

### Data description

We analyze the Temp12k compilation described in ref. 3, to identify age and amplitude of the local HTM and assess uncertainties in reconstructed local HTM characteristics.

In order to avoid bias due to uncomplete coverage of individual records, we first identify records with sufficient coverage by binning individual records into 500-year bins based on their original age model. We choose 500 years because of the minimum 400-year resolution criterion for the dataset selection^3^ and because the broadness of the bins mitigates the influence of chronological uncertainties. The records used in our analyses have at least one value in 80% of the twenty-four 500 year long bins between 0 and 12 ka, thus 20 of the 24 bins. Selected records include 233 (184 annual; 32 summer and 17 winter) marine records and 470 (159 annual; 184 summer and 127 winter) continental records (Fig. 4).

The Temp12k compilation provides age and temperature ensembles (100 realizations each) for 207 of the previously selected marine records and for 462 continental records. Age ensembles were obtained using Bayesian age-depth modeling approach implemented in Bacon^3,36^. Temperature ensembles for pollen data are derived from the Modern Analogue Technique applied by^6^ while temperature ensemble records from marine sediment are based on Bayesian procedures^37–40^ or on multiple generations of analytical and calibration methods (see details in ref. 3). When temperature uncertainties ensemble were not available, we added noise from a standard Gaussian distribution scaled to the temperature uncertainty attributed to each record by the Temp12k consortium to the raw reconstruction (uncertainty on temperature ranges from ±1 to ±3 °C depending on the proxy type according to^3^) and note that these estimates are rather on the conservative side.

In order to characterize the local HTM patterns and to quantify uncertainties associated to ages and temperature reconstruction, we combine all age and reconstruction ensemble members to derive 10,000 realizations of each temperature time series.

### Local HTM analyses

We then identify the maximum temperature for each realization, and obtain its age and amplitude with respect to the Holocene mean. We then use the spread among the 10,000 realizations for each record to quantify the uncertainty in characteristics of the structure of the local HTM.

To analyze the temporal and spatial distribution of the timing of the HTM we use a two-dimensional kernel probability density function (Fig. 5, Gaussian kernel, with a bandwidth of 500 years and 10° in latitude). We calculate the kernel density maps 10,000 using only one realization per records for each iteration (mean shown in Fig. 1). The mean and standard deviation of the resulting two-dimensional PDFs allow to control the robustness of the results and to account for uncertainties in both age and temperature reconstruction. The standard deviation of the PDF remains small relatively to mean PDF values in highest density domain (below 20 and 30% of local PDF for continental and marine records respectively, Fig. 5), suggesting that spatiotemporal patterns discussed in the main text are robust. We also tested other kernel functions (Epanechnikov, box (uniform), triangle) but found no significant differences at the considered bandwidth, suggesting that uncertainty in the data has a larger effect than the choice of kernel. We therefore used a Gaussian kernel with a bandwidth of 500 years in all analyses.

To determine the effect of seasonality and location we repeated our analyses on subsets of the data using one-dimensional kernel probability density function (Gaussian kernel, with a bandwidth of 500 years, Fig. 6). These analyses highlight the robustness of the difference in the timing of the HTM between the continent and the ocean at mid Northern hemisphere latitudes. They also show the effects of seasonality and location in the continental records discussed in the main text.

We assess the effect of proxy sensors on the distribution of HTM ages and amplitude using kernel density-based violin plots (Fig. 7). Despite the large spread among the proxies (which partly reflects regional differences) the early timing of the high latitude marine temperature maxima appears in these plots. Although mean and median HTM age rather occurs around the mid Holocene for both continental and marine environments, marine HTM display a skewed distribution towards the late Holocene, and the mode of the marine HTM often occurs a few thousands of years before the mean and median, and does not result from sensor-specific biases.

Almost all proxy types display few anomalously high amplitudes, which are probably the result of the effect of extraneous variables on temperature estimates. We however have not excluded those from our analyses as their number is too low to significantly affect our results.

### Climate forcing

We show the forcings that apply relatively homogenously over the earth surface (volcanic radiative forcing^41^, decadal solar irradiance changes^42^, and greenhouse gases atmospheric concentrations forcing including pCO_2_^43^, pCH_4_ and pN_2_O^44^) in Fig. 8 and calculate their long-term sum used in Fig. 3. Note that pCO_2_, pCH_4_ and pN_2_O radiative forcing (w/m^2^) were calculated relatively to preindustrial forcing accounting for the overlap in absorption band between N_2_O and CH_4_^45^. Seasonal and monthly insolation anomalies compared to modern insolation for the last 12 ka are displayed in Fig. 9, and mean annual insolation is shown in Fig. 10.

## Data Availability

All data used in this study had previously been published with the TEMP12K database available in LiPD format through WDS-NOAA Paleoclimatology (www.ncdc.noaa.gov/paleo/study/27330; 10.25921/4RY2-G808).

## Code availability

Computer codes are available upon request from O.C.

## References

[1] Mann, M. E. & Jones, P. D. Global surface temperatures over the past two millennia. *Geophys. Res. Lett.*10.1029/2003GL017814 (2003).

[2] Neukom R, Steiger N, Gómez-Navarro JJ, Wang J, Werner JP (2019). No evidence for globally coherent warm and cold periods over the preindustrial Common Era. Nature.

[3] Kaufman D (2020). A global database of Holocene paleotemperature records. Sci. Data.

[4] Kaufman D (2020). Holocene global mean surface temperature, a multi-method reconstruction approach. Sci. Data.

[5] Marcott SA, Shakun JD, Clark PU, Mix AC (2013). A reconstruction of regional and global temperature for the past 11,300 years. Science.

[6] Marsicek J, Shuman BN, Bartlein PJ, Shafer SL, Brewer S (2018). Reconciling divergent trends and millennial variations in Holocene temperatures. Nature.

[7] Liu Z (2014). The Holocene temperature conundrum. Proc. Natl Acad. Sci. USA.

[8] Bova S, Rosenthal Y, Liu Z, Godad SP, Yan M (2021). Seasonal origin of the thermal maxima at the Holocene and the last interglacial. Nature.

[9] Bader J (2020). Global temperature modes shed light on the Holocene temperature conundrum. Nat. Commun..

[10] Park H-S, Kim S-J, Stewart AL, Son S-W, Seo K-H (2019). Mid-Holocene Northern Hemisphere warming driven by Arctic amplification. Sci. Adv..

[11] Lohmann G, Wagner A, Prange M (2021). Resolution of the atmospheric model matters for the Northern Hemisphere Mid-Holocene climate. Dyn. Atmos. Oceans.

[12] Renssen H (2009). The spatial and temporal complexity of the Holocene thermal maximum. Nat. Geosci..

[13] Osman MB (2021). Globally resolved surface temperatures since the Last Glacial Maximum. Nature.

[14] Holland MM, Bitz CM (2003). Polar amplification of climate change in coupled models. Clim. Dyn..

[15] Laepple, T. & Lohmann, G. Seasonal cycle as template for climate variability on astronomical timescales. *Paleoceanography*10.1029/2008PA001674 (2009).

[16] Renssen H, Seppä H, Crosta X, Goosse H, Roche DM (2012). Global characterization of the Holocene Thermal Maximum. Quat. Sci. Rev..

[17] Jonkers L, Kucera M (2017). Quantifying the effect of seasonal and vertical habitat tracking on planktonic foraminifera proxies. Climate.

[18] Jonkers L, Kučera M (2015). Global analysis of seasonality in the shell flux of extant planktonic Foraminifera. Biogeosciences.

[19] Broecker WS (1998). Paleocean circulation during the last deglaciation: a bipolar seesaw?. Paleoceanography.

[20] Shakun JD (2012). Global warming preceded by increasing carbon dioxide concentrations during the last deglaciation. Nature.

[21] Rosell-Melé A, Prahl FG (2013). Seasonality of UK′37 temperature estimates as inferred from sediment trap data. Quat. Sci. Rev..

[22] Rehfeld K, Trachsel M, Telford RJ, Laepple T (2016). Assessing performance and seasonal bias of pollen-based climate reconstructions in a perfect model world. Climate.

[23] Otto J, Raddatz T, Claussen M (2011). Strength of forest-albedo feedback in mid-Holocene climate simulations. Clim. Past.

[24] Patton H (2017). Deglaciation of the Eurasian ice sheet complex. Quat. Sci. Rev..

[25] Ullman DJ (2016). Final Laurentide ice-sheet deglaciation and Holocene climate-sea level change. Quat. Sci. Rev..

[26] Zhang Y, Renssen H, Seppä H (2016). Effects of melting ice sheets and orbital forcing on the early Holocene warming in the extratropical Northern Hemisphere. Clim. Past.

[27] Giesecke T, Brewer S, Finsinger W, Leydet M, Bradshaw RHW (2017). Patterns and dynamics of European vegetation change over the last 15,000 years. J. Biogeogr..

[28] Herzschuh U (2016). Glacial legacies on interglacial vegetation at the Pliocene-Pleistocene transition in NE Asia. Nat. Commun..

[29] Thompson A. J., Zhu J., Poulsen C. J., Tierney J. E. & Skinner C. B. Northern Hemisphere vegetation change drives a Holocene thermal maximum. *Sci. Adv.* **8**, eabj6535 (2022). 10.1126/sciadv.abj6535 PMC9012463 35427164

[30] Sachs, J. P. Cooling of Northwest Atlantic slope waters during the Holocene. *Geophys. Res. Lett.*10.1029/2006GL028495 (2007).

[31] Ayache M, Swingedouw D, Mary Y, Eynaud F, Colin C (2018). Multi-centennial variability of the AMOC over the Holocene: a new reconstruction based on multiple proxy-derived SST records. Glob. Planet. Change.

[32] Jackson L (2015). Global and European climate impacts of a slowdown of the AMOC in a high resolution GCM. Clim. Dyn..

[33] Brierley CM (2020). Large-scale features and evaluation of the PMIP4-CMIP6 midHolocene simulations. Clim. Past.

[34] Otto-Bliesner BL (2017). The PMIP4 contribution to CMIP6 – Part 2: Two interglacials, scientific objective and experimental design for Holocene and Last Interglacial simulations. Geosci. Model Dev..

[35] IPCC. *Climate Change 2014: Impacts, Adaptation, and Vulnerability. Part B: Regional Aspects. Contribution of Working Group II to the Fifth Assessment Report of the Intergovernmental Panel on Climate Change* (Cambridge University Press, 2014).

[36] Blaauw M, Christen J (2011). Flexible Paleoclimate age-depth models using an autoregressive gamma process. Bayesian Anal..

[37] Malevich SB, Vetter L, Tierney JE (2019). Global core top calibration of δ18O in planktic Foraminifera to sea surface temperature. Paleoceanogr. Paleoclimatol..

[38] Tierney JE, Tingley MP (2018). BAYSPLINE: a new calibration for the alkenone paleothermometer. Paleoceanogr. Paleoclimatol..

[39] Tierney JE, Tingley MP (2014). A Bayesian, spatially-varying calibration model for the TEX86 proxy. Geochim. Cosmochim. Acta.

[40] Tierney JE, Malevich SB, Gray W, Vetter L, Thirumalai K (2019). Bayesian calibration of the Mg/Ca paleothermometer in planktic Foraminifera. Paleoceanogr. Paleoclimatol..

[41] Kobashi, T. et al. Volcanic influence on centennial to millennial Holocene Greenland temperature change. *Sci. Rep.* **7**, 1441 (2017). 10.1038/s41598-017-01451-7 PMC5431187 28469185

[42] Wu, C.-J., Krivova, N. A., Solanki, S. K. & Usoskin, I. G. Solar total and spectral irradiance reconstruction over the last 9000 years. *A&A* **620**, A120 (2018).

[43] Indermühle A (1999). Holocene carbon-cycle dynamics based on CO2 trapped in ice at Taylor Dome, Antarctica. Nature.

[44] Flückiger J (2002). High-resolution Holocene N_2_O ice core record and its relationship with CH4 and CO2. Glob. Biogeochem. Cycles.

[45] Joos F, Spahni R (2008). Rates of change in natural and anthropogenic radiative forcing over the past 20,000 years. Proc. Natl Acad. Sci. USA.

[46] Locarnini, M. et al. *World Ocean Atlas 2018, Volume 1: Temperature* (NOAA Atlas NESDIS 81, 2018).

[47] Lambeck K, Rouby H, Purcell A, Sun Y, Sambridge M (2014). Sea level and global ice volumes from the Last Glacial Maximum to the Holocene. Proc. Natl Acad. Sci. USA.

[48] Berger A, Loutre MF (1991). Insolation values for the climate of the last 10 million years. Quat. Sci. Rev..

[49] Berger AL (1978). Long-term variations of caloric insolation resulting from earths orbital elements. Quat. Res..

[50] Huybers, P. & Eisenman, I. Integrated summer insolation calculations. NOAA/NCDC Paleoclimatology Program data contribution. http://eisenman.ucsd.edu/code/daily_insolation.m (2006).

