# Author Correction: Complex spatio-temporal structure of the Holocene thermal maximum

**DOI:** 10.1038/s41467-022-34499-9

**Published:** 2022-11-08

**Authors:** Olivier Cartapanis, Lukas Jonkers, Paola Moffa-Sanchez, Samuel L. Jaccard, Anne de Vernal

**Affiliations:** 1 CEREGE, Aix Marseille Université, CNRS, IRD, INRAE, Coll. France, Technopole Arbois, 13545 Aix en Provence Cedex 4, France; 2 MARUM - Center for Marine Environmental Sciences, University of Bremen, Leobener Straße 8, 28359 Bremen, Germany; 3 Department of Geography, Durham University, Durham, DH1 3LE UK; 4 Institute of Earth Sciences, University of Lausanne, Géopolis, 1015 Lausanne, Switzerland; 5 Oeschger Center for Climate Change Research, University of Bern, 3012 Bern, Switzerland; 6 Geotop, Université du Québec à Montréal, PO Box 8888, Montréal, QC H3C 3P8 Canada

**Keywords:** Palaeoclimate, Palaeoceanography, Climate and Earth system modelling

Correction to: *Nature Communications* 10.1038/s41467-022-33362-1, published online 03 May 2009

The original version of this Article contained an error in Figs. 3 and 8, in which the global forcing curve was calculated and plotted incorrectly with incorrect description of variables in the Figure Caption of Figure 8. This read ‘volcanic radiative forcing^41^; decadal solar irradiance^42^, mean annual insolation at 65°N^48–50^, and relative sea level^47^’ and now reads: ‘volcanic radiative forcing^41^; decadal total solar irradiance variations^42^ at earth surface (ΔTSI at the top of the atmosphere × (1 – albedo)/4, with mean albedo =0.29), mean annual insolation at 65°N^48–50^, and relative sea level^47^’. This has been corrected in both the PDF and HTML versions of the Article.

